# Prevalence and early risk factors for bulimia nervosa symptoms in inner-city youth: gender and ethnicity perspectives

**DOI:** 10.1186/s40337-021-00479-5

**Published:** 2021-10-21

**Authors:** Vladislav Ruchkin, Johan Isaksson, Mary Schwab-Stone, Andrew Stickley

**Affiliations:** 1 Child and Adolescent Psychiatry Unit, Department of Neuroscience, Uppsala University, 751 85 Uppsala, Sweden; 2 Child Study Center, Yale University Medical School, New Haven, CT 06520 USA; 3 Säter Forensic Psychiatric Clinic, 783 27 Säter, Sweden; 4 Department of Preventive Intervention for Psychiatric Disorders, National Institute of Mental Health, National Center of Neurology and Psychiatry, Kodaira, Tokyo, Japan; 5 Stockholm Center for Health and Social Change (SCOHOST), Södertörn University, Huddinge, Sweden; 6 Center of Neurodevelopmental Disorders (KIND), Centre for Psychiatry Research, Department of Women’s and Children’s Health, Karolinska Institutet, Stockholm, Sweden

**Keywords:** Bulimia nervosa, Internalizing problems, Risk factors, Prevalence, Inner-city, Gender, Adolescents

## Abstract

**Background:**

Research on risk factors associated with bulimia nervosa symptoms (BN) in ethnic minorities has been limited. This study adds to the existing literature by providing the ethnicity- and gender-specific prevalence of BN in US inner-city youth and by exploring the longitudinal associations between a clinical level of BN and early risk factors assessed one year previously.

**Methods:**

The study was conducted on a representative sample of predominantly ethnic minority youth (N = 2794; 54.1% female; age 11–16 years old (M(SD) = 12.77(1.29)); 60.0% African-American, 26.1% Hispanic American, 13.9% White). Self-reported information was obtained on BN and early risk factors (e.g., depressive and anxiety symptoms, posttraumatic stress, somatic complaints). Multivariate analysis of covariance was used to examine the longitudinal associations.

**Results:**

The 3-month BN prevalence was higher in girls (5.1%) than in boys (2.3%) (ratio 2.22:1). Significant differences in BN rates were found between White and African American students (higher in Whites), whereas Hispanic-Americans did not differ significantly from either group. Individuals with BN had significantly higher levels of early risk factors one year prior.

**Conclusions:**

Timely recognition of BN and associated early risk factors is important for early prevention and intervention strategies.

**Supplementary Information:**

The online version contains supplementary material available at 10.1186/s40337-021-00479-5.

## Plain English summary

This longitudinal study evaluated the prevalence of bulimia nervosa (BN) symptoms in US inner-city predominantly ethnic minority youth and explored the associations between early risk factors and probable BN one year later. The study was conducted on a large group of inner city youth (N = 2794; 54.1% female; age 11–16 years old (M(SD) = 12.77(1.29)); 60.0% African-American, 26.1% Hispanic American, 13.9% White). Self-reports were used to assess probable BN and early risk factors (e.g., depressive and anxiety symptoms, posttraumatic stress). The 3-month BN prevalence was higher in girls (5.1%) than in boys (2.3%). Significant differences in BN rates were found between White and African American students (higher in Whites), whereas Hispanic-Americans did not differ significantly from either group. Both boys and girls with BN had significantly higher levels of early risk factors as assessed one year previously. Timely recognition of BN symptoms and associated early risk factors is important for early prevention and intervention strategies.

**Supplementary Information:**

The online version contains supplementary material available at 10.1186/s40337-021-00479-5.

## Background

Bulimia nervosa (BN) is a common eating disorder characterized by recurrent episodes of binge eating and compensatory behaviors such as self-induced vomiting to prevent weight gain [34, 81]. Its symptoms are difficult to treat, often chronic [22, 34, 81], and have been associated with a variety of negative psychological, physical and social consequences [45] including an increased risk for mortality [80].

BN symptoms tend to peak in late adolescence [3], but in recent decades have also been more commonly observed in younger children and youth [23, 34, 67]. It has been noted that the general prevalence of dieting and unhealthy weight-control behavior tends to remain fairly consistent in the same individuals from adolescence to young adulthood [46, 66]. However, a substantial proportion of young women report an increase in the severity of BN symptoms over time [70] and physical and mental health problems related to eating disorders tend to be particularly severe during adolescence [17], supporting the notion that early identification of those at risk may potentially improve the course and prognosis of the disorder.

Although BN symptoms have been extensively explored in adolescents [34, 71, 77], many studies have failed to examine gender-specific aspects of BN symptoms and potential associated risk factors, with only a few recent general population studies addressing this important omission (e.g. [29, 42, 43]). While BN symptoms are more common in females (e.g. [77, 43]), rates in males tend to be underestimated [28] and there is a comparative lack of information on disordered eating in boys from the general population (e.g. [56]). Some recent studies suggest that the female to male ratio in adolescents may be as low as 5:1 [29], or even 4:1 [43], as compared with a 10:1 ratio in adults [9]. Exploring BN rates from a gender perspective in the general population, and especially among urban ethnic minority youth, is thus timely and important.

Indeed, even though higher socio-economic status or White race are no longer considered as major risk factors for disordered eating (e.g. [26, 40]), as yet, research exploring the prevalence rates of bulimic symptoms in disadvantaged, ethnic minority youths has been limited [6, 16]. While some studies suggest substantial similarities in the prevalence of eating disorders among different ethnic groups [41], others have found ethnic differences in the prevalence of BN symptoms, suggesting that African-American adolescents [27, 75] and Hispanic-American adolescents and young adults [68, 77] may have higher rates of BN symptoms that Whites. These studies, however, focused predominantly on girls and did not consider potential gender differences within ethnicity. In addition, an accurate assessment of BN prevalence rates may be complicated because ethnic minorities in general are less likely to seek help for eating problems [14], to have such problems inquired about and/or to receive a referral for their further evaluation [7]. Hence, gaining a better understanding of the gender-specific prevalence of BN symptoms in different ethnic groups, in particular, among those with a low socio-economic status, and of the associated early risk factors, is vitally important.

Problems with mental health have long been considered among risk factors associated with BN, from both cross-sectional and longitudinal perspectives. Indeed, psychiatric comorbidity in eating disorders in adolescents is frequent (e.g. [77]), being particularly high in clinical settings [30], most commonly of anxiety and depression [32, 35, 84], posttraumatic stress [77], and substance abuse [1]. Both early depressive symptoms [44, 84] and negative affectivity (e.g. [72]) may precede the onset of BN symptoms in youth. Comorbid anxiety disorders are also common in relation to BN [58], and BN further increases the prospective risk for anxiety and depressive disorders, drug use and self-harm [42]. In addition to mental health risk factors associated with BN in youth, previous research has also identified a greater prevalence of somatic symptoms, such as gastrointestinal complaints, and problems in cardiovascular and endocrine systems as being important in this context [30]. Moreover, it has been suggested that some personality traits, such as sensation seeking [25, 82] and its impulsivity component [38], may play a significant role in BN. Although these multiple risk factors often coexist, as yet, few studies have tried to evaluate their role in the same group of individuals, making it difficult to disentangle the specific effect of each particular symptom group. To our knowledge, no previous study has attempted to evaluate whether these risk factors may also be gender- or ethnicity-specific.

Hence, there is a need to further investigate the prevalence of eating problems in large ethnically diverse adolescent populations, especially using a gender perspective, while also assessing potential risk factors, which could help to facilitate prevention efforts.

The aims of this study therefore were to: (1) determine the gender- and ethnicity-specific prevalence of BN symptoms in a representative sample of inner-city ethnic minority youth, (2) explore whether adolescents with probable BN are more likely to have reported risk factors (depression, anxiety, posttraumatic stress, somatic symptoms and sensation seeking) a year earlier and whether these effects are moderated by gender and ethnicity. We will examine a range of mental health risk factors that have been theorized to increase the risk for BN in several previous studies. Considering that these problems are often comorbid and overlap, it is important to investigate how the multitude of these problems may be associated with BN symptoms over time in the same analysis, to explore whether individuals with possible BN differ from others in terms of early comorbid problems and whether these patterns may be to some extent gender- and ethnicity-specific.

Based on previous research findings, we hypothesized that girls would report higher rates of BN symptoms than boys, but the gender ratio of BN may be lower than that reported in earlier studies. We did not make any specific hypothesis regarding BN rates in different ethnicities, given the substantial diversity of the results in previous reports. We further hypothesize that adolescents with BN will report higher levels of risk factors one year earlier and that these effects may be gender- and ethnicity-specific.

## Methods

### Participants

The survey was administered to all eligible students in the New Haven (CT) public school system, including students in alternative programs and bilingual classes (17 public middle and high schools). In the spring of year 1, 3562 11–16 year old students completed the survey. This sample was followed until the next survey administration one year later. Eighty percent of the original sample (n = 2847) completed the survey in year 2. High attrition rates in longitudinal studies of urban ethnic minority adolescents are common (e.g., [49]), related to the high mobility of the families and elevated rates of school dropout. Results indicated that the 715 students who dropped out (compared to the 2847 remaining students) were older (t = 14.38, *p* < .001) and more likely to be male (428 (59.9%) vs. 1310 (46.0%), χ^2^ = 4.09, *p* < .05). These drop-out students reported higher levels of depression (M (SD) = 5.22 (4.60) vs. 4.67 (4.19), t = 3.16, d = .150, *p* < .01) and posttraumatic stress (M (SD) = 22.96 (13.94) vs. 21.76 (13.07), t = 2.27, d = .089, *p* < .05). These findings suggest that attrition was selective across several key variables.

To examine ethnicity as a variable in analyses, the sample was restricted to participants with an African-American, Hispanic-American, and White ethnic background, which resulted in the exclusion of 53 (1.9%) subjects from other ethnic groups (i.e., Asian-American and Other).

Finally, 168 adolescents had missing data. In relation to this, multiple imputation was undertaken (i.e., data were imputed 20 times) using the Markov Chain Monte Carlo (MCMC) method with a Predictive mean matching (PMM) model for continuous variables. No categorical background variables lacked data, as information on these variables was obtained from the school registry.

The final sample (N = 2794; 54.1% female; age at baseline 11–16 years (M(SD) = 12.77(1.29)) (11yo n = 424; 12yo = 916; 13yo = 659; 14yo = 394; 15yo = 326; 16yo = 75) was predominantly comprised of minority ethnicities (60.0% African-American, 26.1% Hispanic American, 13.9% White), an accurate reflection of the local public school population [47]. A majority of the students came from single parent families (54.3%). The sample population was socio-economically disadvantaged overall, as reflected by the large proportion (over 71%), who qualified for free/reduced lunch status at either point of the data collection. Over eighty percent of students’ mothers and fathers had the equivalent of a high school education or beyond.

### Procedure

Parents were informed of the survey at the time of school registration, received a letter about the survey two weeks prior to its administration, and were offered the opportunity to decline participation. The passive informed consent procedure was approved by the university’s institutional review board and considered as an appropriate ethical procedure by the state legislature. Prior to survey administration, students were read a detailed assent form outlining their participation with the assurance of confidentiality, and were asked to sign it to indicate assent (parent and child refusals were less than 1%). Students completed the survey in a classroom setting during one class period during a regular school day. Trained administrators read all the questions aloud while students followed along with their copies of the survey, reading questions to themselves and circling responses in the booklet. A second administrator was available, providing help to individual students if requested. Surveys were administered in English and Spanish. All students in the respective grades attending school on the day of the survey’s administration were eligible to participate. Make-up administrations were performed at each school within one month of the initial administration for those who had been absent.

### Measures

This study is part of an ongoing project (the Social and Health Assessment, SAHA) that aims to assess risk and protective factors for adolescent adjustment, which has been administered annually in collaboration with the public school system in New Haven (CT) for nearly two decades. The survey has been extensively used in previous research and includes both scales available from the literature that have been used with similar populations and a few new scales developed specifically for this study, which have been subsequently validated against the existing scales in this particular study group [61].

### Measures in Year 1

*Depressive symptoms* were assessed with an adaptation of the *Center for Epidemiologic Studies-Depression Scale* (CES-D; [54]). Studies of adolescent populations have shown that the CES-D (e.g. [57]) and its modified versions (e.g. [10]) have excellent psychometric properties. The scale consists of 10 negative statements (e.g. “I felt like crying”; “I felt that many bad things were my fault”), but an item on poor appetite was removed for this study to avoid potential overlap with the eating problems. The presence of depressive symptoms during the past month was assessed using a three-point scale (“Not true” (scored 0); “Somewhat true” (1); or “Certainly true” (2)). The total scale score ran from 0 to 18 with higher scores indicating more depressive symptoms. McDonald’s omega was .82 for the African-Americans, .84 for Whites and .81 for Hispanic-Americans.

*Anxiety symptoms* were measured with a 12-item scale [61] which included questions on worrisome or preoccupying thoughts and feelings (e.g. “I feel nervous when I get called on in class”, “I stay away from things that make me nervous”). The measure was developed for the SAHA, validated against the BASC anxiety scale [61] and has been used extensively in previous studies (e.g. [62, 63]). The above-mentioned three-point answer scale was chosen to provide uniformity when assessing internalizing problems (“Not true” (scored 0), “Somewhat true” (1); or “Certainly true” (2)). The total score ranged from 0 to 24, with higher scores reflecting increasing anxiety symptoms. McDonald’s omega was .83 for the African-Americans, .88 for Whites and .87 for Hispanic-Americans.

*Posttraumatic stress* was assessed with the Child Post-Traumatic Stress - Reaction Index (CPTS-RI, [51]), a 20-item scale assessing the frequency of posttraumatic stress symptoms on a 5-point scale, ranging from “Never” (0) to ”Most of the time” (4). Higher CPTS-RI scores correspond closely with a clinical diagnosis of posttraumatic stress disorder (PTSD) [52]. The total score could range from 0 to 80 with higher scores indicating greater posttraumatic stress. McDonald’s omega was .92 for the African-Americans, .89 for Whites and .84 for Hispanic-Americans. *Somatic complaints* were examined using a 10 item-scale that assesses commonly occurring somatic complaints in children and adolescents [78]. The presence of somatic symptoms (e.g. “Felt health should be better”, “Had headaches”, “Had nausea”) during the past month was reported on a three-point scale (“Not true” (scored 0); “Somewhat true” (1); or “Certainly true” (2)). The total score could range from 0 to 20; higher scores reflecting increasing levels of somatic complaints. McDonald’s omega was .79 for the African-Americans, .83 for Whites and .80 for Hispanic-Americans. *Sensation seeking* was assessed with the Brief Sensation Seeking Scale (BSSS, [33]). This measure consists of 8 items describing thrill and excitement seeking, disinhibition and boredom susceptibility (e.g. “I would like to explore strange places,” “I like to do frightening things”). Responses are rated on a 5-point scale, ranging from “Strongly disagree” (0) to “Strongly agree” (4). The total score could range from 0 to 32; higher scores indicating higher levels of sensation seeking. McDonald’s omega was .79 for the African-Americans, .81 for Whites and .81 for Hispanic-Americans. *Proxy for socioeconomic status (SES)*. Eligibility for free (2) or reduced lunch (1) was used as an index of SES and could hence vary between 0 and 2. Students were eligible if their family’s income was less than 185% of the federal poverty threshold.

### Measures in Year 2

*Disordered eating behaviors* were assessed using a shortened version of the Eating Disorder Diagnostic Scale [69], which measures disordered eating thoughts and behaviors in the past 3-months. It consists of four statements on the occurrence of bulimia/anorexia symptoms (described below), to be rated as “Not true” (0), “Somewhat true” (1), and “Certainly true” (2). The internal consistency of this scale was good (Cronbach’s α = 81). In addition, two questions assessed the frequency (per week) of compensatory behaviors to prevent weight gain on a 5-point scale ranging from “0 times” (scored 0) to “More than 10 times” (4).

A proxy for *a clinical level of BN symptoms* (probable BN) was created using DSM-5 criteria for BN [3]. Diagnostic criterion A (recurrent episodes of binge eating) was coded based on the item: I ate large amounts of food, even when I didn’t feel hungry. B and C criteria (recurrent inappropriate compensatory behaviors, such as the use of laxatives, vomiting and fasting or excessive exercise, at least once a week for 3 months that are intended to prevent weight gain) were assessed with two items: About how many times per week have you made yourself vomit or used laxatives to prevent weight gain? (at least once) OR About how many times per week have you fasted (skipped at least 2 meals in a row) or engaged in excessive exercise to prevent weight gain? (at least once). Criterion D (body shape and weight unduly influence self-evaluation) was categorized based on a positive response to any of the following statements: I felt fat even when others told me I am too thin, OR I felt very upset about my overeating or weight gain, OR I worried a lot about how to stop gaining weight. Positive symptom scores for all four diagnostic criteria (Certainly true responses only on the occurrence of BN symptoms, at least once a week responses for any of the compensatory behaviors) were used to create a binary variable (1/0), which was used in all analyses and denoted probable BN (stringent definition). In addition, a less stringent definition of probable BN was also examined (in the prevalence analyses only), using both Certainly true and Somewhat true ratings for BN symptoms, but the same frequency of compensatory behaviors.

### Statistical analyses

The Statistical Package for the Social Sciences (SPSS-25.0) was used to analyse data. Chi-square (with Cramer’s V) and independent sample t-tests (with Cohen’s d effect sizes) were performed when making univariate comparisons of demographic characteristics and comparing the prevalence of probable BN by ethnicity and SES. For chi-square tests with more than two groups (i.e. ethnicity), pair-wise tests (z-tests with Bonferroni adjustments) were run in order to determine if there were any differences between the groups. Pearson correlation coefficients were calculated between the outcome variables. General linear models (GLM) multivariate analysis of covariance (MANCOVA) was used to determine main and interaction effects across the fixed factors of probable BN (1/0) (as described earlier), gender (girls = 0, boys = 1), and ethnicity (African-American (1), White (2), and Hispanic American (3)), while adjusting for the SES and age covariates. MANCOVA analyses were chosen over ANCOVAs because evidence suggests that these risk factors commonly co-occur (e.g. [60]) and thus there is a need to disentangle the individual effects of each particular risk, while adjusting for confounders. However, a series of separate Uni-ANCOVAs were later conducted for each particular outcome.

MANCOVA analyses were conducted with potential risk factors reported one year prior to the assessment of probable BN (including depression and anxiety symptoms, somatic complaints, posttraumatic stress, and sensation seeking). Thus, we used a 2 (probable BN) X 2 (gender) X 3 (ethnicity) design for assessing differences in early risk factors at year one. The unique contribution of each of the three fixed factors, the two covariates (SES proxy, age), and the four interaction terms were assessed through follow-up between-subject tests and unstandardized parameter estimates derived from the MANCOVA. Results are presented as means (*M*) and standard deviations (*SD*), and for individual outcomes, as Wilks' lambda, F(df), p and partial eta squared (η^2^), a common metric of effect size that represents the unique amount of variance explained by each predictor variable. Cohen [15] provided points of reference to define small (η2 = .01), medium (η2 = .06), and large (η2 = .14) effects.

## Results

### Gender differences in BN symptoms and early risk factors

The prevalence of BN symptoms by gender within each ethnic group (*%*) is presented in Table 1. The prevalence of individual BN symptoms varied greatly from 2.9% (for feeling fat even when others told me I am too thin (in Hispanic-American boys)), to 40.1% (for fasting or engaging in excessive exercise to prevent weight gain (in Hispanic-American girls)). Most BN symptoms were significantly more prevalent in girls than boys in all ethnic groups, except for compensatory behaviors, which did not differ by gender among the three ethnicities (except for fasting or engaging in excessive exercise to prevent weight gain, which was higher in Hispanic-American girls than boys). The prevalence of probable BN in the whole study group also differed by gender (2.3% in boys vs. 5.1% in girls, chi-square = 15.60; Cramer’s V = .074; *p* < .001, using the stringent criteria of probable BN, or 8.2% in boys vs. 13.4% in girls, chi-square = 19.81; Cramer’s V = .083; *p* < .001, when using the less stringent probable BN criterion).

**Table 1:** Prevalence of disordered eating behaviors by ethnicity and gender (%)

During the past three months	African-American		Chi-square, Cramer’s V; *p*	White		Chi-square; Cramer’s V; *p*	Hispanic- American		Chi-square; Cramer’s V; *p*
	Boys (n = 815)	Girls (n = 902)		Boys (n = 177)	Girls (n = 192)		Boys (n = 344)	Girls (n = 364)	
*Disordered eating symptoms/behaviors*									
I worried a lot about how to stop gaining weight	7.9	21.0	58.49; .185; < .001	15.3	32.3	14.61; .199; < .001	11.3	29.4	35.23; .223; < .001
I felt fat even when others told me I am too thin	3.9	14.9	58.56; .185; < .001	6.8	17.7	10.08; .165; < .001	2.9	23.9	65.93; .305; < .001
I ate large amounts of food even when I didn’t feel hungry	9.7	15.1	11.33; .081; < .001	11.3	11.5	.002; .002; .962	6.7	14.8	12.12; .131; < .001
I felt very upset about my overeating or weight gain	5.5	13.7	31.73; .138; < .001	9.0	21.4	10.69; .170; < .01	7.8	21.4	25.82; .191; < .001
*Compensatory behaviors*									
I made myself vomit or used laxatives to prevent weight gain	6.1	6.9	.424; .016; .515	7.3	8.3	.124; .018; .724	8.7	9.6	.170; .015; .680
I fasted (skipped at least 2 meals in a row) or engaged in excessive exercise to prevent weight gain	27.2	28.0	.140; .009; .708	27.7	34.9	2.22; .078; .136	28.8	40.1	10.03; .119; .002

Comparisons are made by gender within each ethnicity. Prevalence is given for *certainly true* responses for the occurrence of disordered eating behaviors and at least *once a week* responses for the compensatory behaviors

When comparing the other outcome variables by gender, boys (as compared to girls) reported lower levels of depressive symptoms, *M* (*SD*s) = 3.65 (3.08) vs. 5.11 (3.83), *t* = 11.04, d = .426, *p* < .001; anxiety *M* (*SD*s) = 8.74 (5.54) vs. 10.19 (5.44), *t* = 6.88, d = .270, *p* < .001; somatic complaints *M* (*SD*s) = 5.67 (4.59) vs. 7.15 (4.88), *t* = 8.19, d = .312, *p* < .001, and posttraumatic stress *M* (*SD*s) = 19.85 (12.44) vs. 23.57 (13.49), *t* = 7.50, d = .291, *p* < .001, and higher levels of sensation seeking *M* (*SD*s) = 5.69 (2.10) vs. 4.56 (2.21), *t* = 13.79, d = .525, *p* < .001.

### Probable BN in relation to ethnicity and SES

The prevalence of probable BN differed significantly by ethnicity, with the highest prevalence reported by Whites, followed by Hispanic-Americans and by African-Americans (Table 2), but significant differences were only observed between Whites and African-Americans. When using the less stringent definition of probable BN, the prevalence was highest in Hispanic-Americans, followed by Whites and by African-Americans, but the only significant difference was observed between African-American and Hispanic-American students. When assessing probable BN by gender between the ethnic groups, its prevalence in girls (using the more stringent criterion) did not significantly differ by ethnicity, whereas when a broader criterion of probable BN was used, a significant difference was again observed between African-American and Hispanic-American students. Regarding the prevalence rates of probable BN (stringent criterion) in boys, Whites had significantly higher rates, as compared to African-Americans and Hispanic students (Table 2), but the rates did not differ significantly when applying less stringent criteria for probable BN. These comparisons in boys seem however somewhat inconclusive, considering that there was only a small number of subjects in each cell for BN. No significant differences were observed, when comparing probable BN by the SES proxy (qualified for free and reduced lunch vs. others) (chi-square = 1.088, *p* = .297).

**Table 2:** Prevalence of probable BN by ethnicity and gender (%)

	Ethnicity			Comparisons by ethnicity
	African-American (n = 1717)	White (n = 369)	Hispanic-American (n = 708)	(Chi-square, Cramer’s V, *p*)
*Probable BN*				
Boys	1.8 ^a^	5.6 ^b^	1.5 ^a^	8.39; .090; .004
Girls	4.2	6.8	6.6	4.21; .054; .122
*Comparisons by gender (Chi-square, Cramer’s V, p)*				
	7.28; .068; .007	.198; .023; .656	10.89; .130; < .001	
Total	3.1 ^a^	6.2 ^b^	4.1 ^a,b^	8.61; .056; .013
*Probable BN, less stringent*				
Boys	7.5	9.6	8.7	1.13; .029; .568
Girls	11.3 ^a^	14.1 ^a,b^	18.4 ^b^	11.30; .088; .004
*Comparisons by gender (Chi-square, Cramer’s V, p)*				
	7.29; .065; .007	1.74; .069; .187	14.03; .141; < .001	
Total	9.5 ^a^	11.9 ^a,b^	13.7 ^b^	9.63; .059; .008

A same subscript letter denotes categories whose column proportions do not differ significantly from each other at the .05 level

### Probable BN and early risk factors

#### MANCOVA main effects

When evaluating the differences in early risk factors by probable BN (see Table 3 for descriptive statistics (*M* (*SD*)) by gender and Table 4 for the main and interaction effects, and for the tests of between-subjects effects) the main effect for the model was significant (Wilks’ lambda = .808; *F* (5, 2776) = 131.51, *p* < .001, η^2^ = .192). With regard to specific effects, the main effect for probable BN was significant, with higher levels of all early risk factors (except for sensation seeking) in those with probable BN. The main effect for Gender was also significant, demonstrating higher levels of all early internalizing risk factors (except for anxiety) among girls and sensation seeking in boys, see Tables 3 and 4. The main effect for Ethnicity was not significant, suggesting no difference in early risk factors by ethnic status. The main effect for the SES proxy (free lunch) was significant, suggesting differences in early risk factors by SES, and more specifically in depression, somatic complaints and posttraumatic stress. The main effect for Age was also significant and, as suggested by the follow-up tests, was related to higher anxiety levels along with increasing age.

**Table 3:** Early risk factors (M (SD)) by ethnicity and probable BN^†^ in boys (B) and girls (G)

	African-American (n = 1717)		White (n = 369)		Hispanic American (n = 708)	
	Probable BN	No BN	Probable BN	No BN	Probable BN	No BN
*Depressive symptoms*						
B	4.40 (3.96)	3.64 (2.93)	3.50 (3.21)	3.15 (3.17)	7.40 (5.03)	3.77 (3.41)
G	7.00 (4.25)	4.92 (3.72)	6.46 (5.58)	5.00 (3.85)	6.83 (4.54)	5.25 (3.83)
*Anxiety symptoms*						
B	11.07 (6.54)	8.75 (5.31)	8.30 (5.38)	7.81 (5.69)	15.40 (5.41)	9.14 (5.98)
G	12.27 (6.33)	9.67 (5.26)	10.77 (6.65)	10.43 (5.46)	11.46 (5.49)	10.93 (5.57)
*Somatic complaints*						
B	5.20 (4.44)	5.58 (4.42)	6.30 (4.35)	4.36 (3.83)	11.20 (3.03)	6.31 (5.15)
G	10.43 (5.80)	6.73 (4.69)	9.92 (5.50)	6.97 (5.11)	8.63 (4.27)	7.62 (4.85)
*PTS*						
B	24.80 (12.23)	20.34 (11.87)	17.50 (10.33)	16.85 (12.13)	39.00 (16.31)	19.45 (13.16)
G	32.14 (14.97)	23.22 (13.28)	28.62 (17.43)	22.73 (14.41)	26.63 (12.01)	23.51 (12.85)
*Sensation seeking*						
B	5.80 (2.40)	5.65 (2.00)	5.20 (2.49)	6.20 (2.25)	6.40 (1.82)	5.63 (2.13)
G	4.97 (1.64)	4.56 (2.18)	5.77 (2.05)	4.07 (2.12)	5.62 (2.00)	4.72 (2.36)

Probable BN^†^—Probable Bulimia Nervosa; PTS – Posttraumatic stress

**Table 4:** Main and interaction effects and effect sizes for each dependent variable (early risk factors) (η^2^, p)

	Main and interaction effects (Wilks’ lambda, F(df), η^2^, p)	Depressive symptoms	Anxiety symptoms	Somatic complaints	Posttraumatic stress	Sensation seeking
Age	.984; 8.97 (5, 2776); .016; *p* < .001	.001, ns	.008, < .001	.000, ns	.001, ns	.000, ns
Free lunch	.987; 7.11 (5, 2776); .013; *p* < .001	.006, < .001	.001, ns	.007, < .001	.009, < .001	.000, ns
Gender	. 988; 6.72 (5, 2776); .012; *p* < .001	.005, < .001	.001, ns	.004, < .01	.002, < .05	.004, < .01
Probable BN†	.991; 4.82 (5, 2776), .009; *p* < .001	.005, < .001	.003, < .01	.005, < .001	.006, < .01	.001, ns
Ethnicity	996; 1.17 (10, 5552); .002; *ns*	.001, ns	.002, ns	.001, ns	.002, ns	.000, ns
Probable BN† x Gender	.997; 1.42 (5, 2776); .003; *ns*	.000, ns	.001, ns	.000, ns	.000, ns	.002, < .05
Probable BN† x Ethnicity	.997; .83 (10, 5552); .001; *ns*	.001, ns	.001, ns	.000, ns	.001, ns	.000, ns
Gender x Ethnicity	995; 1.40 (10, 5552); .003; *ns*	.001, ns	.001, ns	.003, < .05	.003, < .01	.000, ns
Probable BN† x Gender x Ethnicity	.994; 1.74 (10, 5552); .003; *p* = .048	.001, ns	.001, ns	.003, < .05	.003, < .05	.002, ns

Probable BN†—Probable Bulimia Nervosa

#### MANCOVA interaction effects

As concerns the interaction effects, the interaction effect for probable BN x Gender was not significant, suggesting that the associations of early risk factors with probable BN for the whole group were not gender-specific. The interaction effect for probable BN x Ethnicity was not significant and neither was the interaction effect for Gender x Ethnicity. Finally, the interaction effect for probable BN x Gender x Ethnicity was weakly significant, suggesting gender-specific associations between early risk factors and probable BN within ethnic groups. The follow-up tests showed differences in somatic complaints and posttraumatic stress in relation to probable BN by gender and by ethnicity, with Hispanic-American boys reporting much higher levels of these symptoms than their female counterparts in relation to probable BN, whereas other ethnic groups had an opposite symptom pattern by gender.

#### Uni-ANCOVA

The size of the correlations between the outcome variables varied from low (e.g. between depression and sensation seeking, *p* = .053) to high (e.g. between depression and posttraumatic stress, *p* = .535). As outcome differences might have been obscured by use of the MANCOVA (i.e. by simultaneously assessing several inter-correlated outcomes in one model), in an additional sensitivity analysis, each outcome was examined separately using Uni-ANCOVA in order to determine whether the results that were obtained from the MANCOVA were the same for each individual risk factor. The results obtained were largely the same as those from the MANCOVA analysis (Additional file 1: Table S1). We have also included comparisons of the early risk factors scores by ethnicity and gender in the form of graphs (Additional file 2: Figures 1-5)

## Discussion

This study assessed the 3-month gender- and ethnicity-specific prevalence of probable BN in a large general population sample of urban, predominantly ethnic minority adolescents. Significant differences in probable BN rates were found by ethnicity. Both boys and girls with BN had significantly higher levels of early risk factors one year prior and while the associations were neither gender- nor ethnicity-specific for the whole group, the results indicated possible weak gender-specific effects within a particular ethnicity (Additional file 2).

We found that a substantial number of ethnic minority inner-city youths had experienced BN symptoms during the past 3 months and that the prevalence of the symptoms differed by gender and by ethnicity. When using a more stringent criterion (certainly true symptom ratings) the prevalence of probable BN in the total sample was 5.1% in girls versus 2.3% in boys (2.22:1 ratio). The lifetime prevalence rates of a clinical diagnosis of BN in a nationally representative US sample were estimated at 1.5% in females and .5% among males [34], while the lifetime prevalence in adolescents was slightly lower [77]. Considering that the lifetime prevalence of eating disorders not otherwise specified (EDNOS, consisting primarily of subclinical anorexia and BN symptoms) is estimated to be between .8% and 14%, depending on the definition used [11], this suggests that the prevalence of probable BN in this population, while higher than the diagnostic national estimates, seems more reasonable. Corroborating this, in a large population-based Australian sample, 4.6% of adolescents were recently found to have probable BN (7.7% in females, 1.8% in males, with a gender ratio of 4.27:1) [43].

When applying a broader, less stringent inclusion criterion (somewhat or certainly true symptom ratings, but the same frequency of compensatory behaviors) for probable BN, substantially higher prevalence estimates (13.4% in girls vs. 8.2% in boys) were obtained. Some studies with dimensional measures have estimated the prevalence of disordered eating behaviors as ranging between 14 and 22% [31, 36], while others have suggested that disordered behaviors or attitudes characteristic of eating disorder are substantially more common in adolescents and young adults than the diagnostic prevalence may suggest [53, 55, 71]. The present study supports these findings and suggests that there may be relatively high levels of subthreshold BN symptoms in the adolescent population. Considering that the rates of medical and psychological complications in eating problems that do not fit into a specific diagnostic frame are similar to those for full-threshold disorders [20, 30, 50], gaining a better understanding of individuals who may have clinically significant symptom levels, but who may not seek help is an important task, especially as many of them may have comorbid problems.

It has been recently concluded that there is a general lack of population-based data on disordered eating behaviors among adolescent males [28, 43], who have been described as “underdiagnosed, undertreated and misunderstood” [76]. It is well documented that females have a higher risk of developing BN symptoms [9], and yet, the female-to-male ratio estimates reported in previous studies have varied drastically from 3:1 [77] to 10:1 [37, 41]. Furthermore, the hospitalization rates of males with eating problems have increased sharply in recent decades (by as much as 37%) [2], and younger patients diagnosed with eating disorders are more likely to be boys, as compared to in the older age groups [9]. Our findings of a higher prevalence of probable BN in girls, as compared to boys, is in line with earlier research [41, 43, 64, 77]. At the same time, the prevalence of BN symptoms in males in many previous studies may have been underestimated, especially considering that the rates of compensatory behaviors aimed at preventing weight gain were rather similar by gender in this study. Indeed, some authors have suggested that the gender difference in compensatory behaviors may not be very large, such as in an earlier study where 9% of female and 4% of male high school students reported daily self-induced vomiting, and that many males may therefore have disordered eating behaviors severe enough to warrant medical evaluation [5].

Accumulating evidence suggests that adolescents from ethnic minorities may have higher rates of eating disorders than previously reported [59]. For example, Striegel-Moore et al. [75] found that African-American girls had significantly higher BN scores than White girls, but only in younger age groups (11 to 14 years), whereas middle adolescents (15 and 16 years) scored similarly. Some other studies have also reported higher levels of binge eating in African-American girls, as compared to Whites in middle school [13] and college students [12] and of higher rates of BN symptoms in Hispanic-American adolescents, compared to Whites [68], [77]. In the present study, significantly higher BN rates were found in White students, as compared to African-Americans, whereas Hispanic-Americans did not differ significantly from either group. It should also be noted that the gender ratio varied widely within each ethnic group and that when applying less stringent criteria for probable BN (subthreshold symptoms) the symptom levels in ethnic minorities (both African-Americans and Hispanic-Americans) became substantially higher, both decreasing the gender ratios within the ethnic groups and diminishing differences between ethnicities within each gender.

Our finding that there was no significant association between SES and probable BN does not support previous reports of an inverse relationship between these variables. For example, a study that used data from a large racially mixed sample of high school students found that those in the highest SES group had the lowest levels of binge eating and vomiting [74]. This seeming contradiction may however be explained by the predominantly low SES in the present study group, where over 71% of the students qualified for free/reduced lunch status at either point of the data collection. At the same time, while there may not be differences in eating disorder prevalence based on wealth, there may well be in access to eating disorder treatments as previous studies have emphasized the lack of access to treatment for ethnic minority youth, as well as for those with low SES (e.g. [27]).

In the present study, probable BN was significantly associated with almost every early risk factor, and in particular, posttraumatic stress, somatic and depressive symptoms, even though the levels of association were rather low. When considering predisposing psychiatric symptoms more specifically, past traumatic experiences have long been considered a significant, albeit non-specific, risk factor for disordered eating behaviors [8]. Studies of adults [18, 34] and adolescents [77] alike have reported higher rates of PTSD in individuals with BN. A number of studies have emphasized that adolescents with eating disorders often suffer from primary anxiety and depressive disorders that began before the onset of their eating disorders [35, 84], that may increase the likelihood of overeating and binge eating at follow up by as much as two times [65]. The lack of an interaction effect between probable BN and gender with regard to psychiatric comorbidity suggests that early internalizing problems have a similar, non-gender-specific influence on BN symptoms over time. This is in line with previous reports suggesting that males and females report similar levels of overall psychological distress and reduced quality of life associated with disordered eating behaviors [79]. At the same time, significantly higher levels of early somatic complaints and of posttraumatic stress in Hispanic-American boys, as compared to girls (opposite to the pattern in Whites and African-Americans), in relation to BN suggest that some early risks may be ethnicity-specific. In contrast to this, the levels of internalizing problems in adolescents in general had gender-specific differences, with boys reporting lower levels of internalizing problems than girls, which is similar to what has been observed in previous studies (e.g. [48]).

Early psychiatric morbidity is considered as a general predisposing factor for BN symptoms [83] and patients with disordered eating behaviors may be more vulnerable to stress, as well as exhibit high levels of anxiety sensitivity [8]. Individuals with BN symptoms tend to experience self-awareness difficulties in relation to their own emotional states, as well as with using appropriate strategies to modulate their emotional reactions and to inhibit impulsive behavior in the context of emotional distress [83], leaving them more susceptible to emotional distress and other problems, which may become apparent at a rather early stage, as suggested by the present study. These early psychological conditions associated with BN symptoms may also substantially influence the degree of functional impairment observed in disordered eating behaviors, hence further complicating the situation [19]. Individuals with BN symptoms have also been shown to engage in increased sensation seeking behavior [25] and have elevated levels of impulsivity/urgency [24], and it has been argued that binge eating might be used to cope with the high levels of negative affect that may arise from such personality traits [73]. However, despite this earlier research, in the present study we did not find an association between sensation seeking and probable BN, which highlights the importance of continuing to examine these relations in samples stratified by various factors such as gender and ethnicity in future studies.

Before concluding, several study limitations should be mentioned. First, although the study showed a longitudinal association between early psychological and behavioral problems and subsequent probable BN, it was not possible to establish causality. In this respect, being able to control for baseline levels of BN symptoms would have been beneficial. Second, the use of students’ self-reports means that the data may be subject to different forms of bias such as social desirability bias, and recall and reporting biases. Third, the study did not inquire specifically about whether the students had been previously diagnosed with or received any treatment for BN or any other eating disorder and other mental health problems. In addition, the study did not assess whether BN symptoms were associated with any functional impairment or problems with overweight. This is important, as it is likely that some of the adolescents, who were categorized as having probable BN, would not meet the diagnostic criteria for BN in clinical terms, making the results less generalizable to clinical populations. The three items (feeling fat when others say I am too thin; feeling very upset about overeating or weight gain; and worrying a lot about how to stop gaining weight) chosen for the assessment of Criterion D (body shape and weight unduly influence self-evaluation) may have not been able to operationalize the concept accurately, considering that BN symptoms may be even more common in those who are overweight (e.g. [42, 43]). This will have reduced the prevalence of BN in this study. Finally, the study had high attrition rates (80% of the original study sample retained), typical for urban, ethnic minority, low-income populations, as they tend to move frequently [39], may be forced to leave the city because of gentrification [4] and especially due to the fact that they have elevated school dropout rates that can reach up to 50–80% in some metropolitan areas [21]. Hence, school-based research projects with ethnic minority students are likely to encounter substantial attrition among the highest-risk youths, limiting the generalizability of findings. Indeed, the students that dropped out in the present study reported higher levels of comorbid problems than those in the follow-up group and hence, they potentially represent a more vulnerable population and their inclusion in the study could have impacted the results.

## Conclusions

Similar to previous research with adolescents from the general population, ethnic minority economically disadvantaged youth from the present study reported a substantial number of BN symptoms. Adolescents with BN may already differ from others on a range of risk factors at a relatively early, preclinical stage, regardless of gender or ethnicity. Timely recognition of BN symptoms and associated early risk factors is important for early prevention and intervention strategies, which should be implemented regardless of background, ethnicity and gender. Future longitudinal research on large adolescent general population samples is needed in order to get a better understanding of the rates of BN and their dynamics over time by gender and in different ethnic groups.

## Supplementary Information

**Additional file 1**.** Table S-1**. Statistics for ANCOVA tests, conducted separately for each dependent variable (early risk factors) (F(1,2794), η2, p).

**Additional file 2**.** Figures 1–5**. Comparisons of the early risk factors scores by ethnicity and gender.

## Data Availability

Access to the data is restricted to the research group members in accordance with the initial IRB decision.

## Abbreviations

BN: Bulimia nervosa
PTSD: Post-traumatic stress disorder
