# Supplementary material for: Prevalence and early risk factors for bulimia nervosa symptoms in inner-city youth: gender and ethnicity perspectives
Source: J Eat Disord. 2021 Oct 21;9:136. doi: 10.1186/s40337-021-00479-5 (PMC8529812; doi:10.1186/s40337-021-00479-5)
Supplement: Supplementary file 1 — Additional file 1. Table S-1. Statistics for ANCOVA tests, conducted separately for each dependent variable (early risk factors) (F(1,2794), η2, p). [file 40337_2021_479_MOESM1_ESM.docx]

Table S-1. Statistics for ANCOVA tests, conducted separately for each dependent variable (early risk factors) (F(1,2794), η^2^, p)

|  | Depressive symptoms | Anxiety symptoms | Somatic complaints | Posttraumatic stress | Sensation seeking |
| --- | --- | --- | --- | --- | --- |
| Intercept | 74.61, .027, p<.001 | 317.70, .103, p<,001 | 138.30, .047, p<.001 | 222.90, .074, p<.001 | 306.23, .099, p<.001 |
| Age | 3.03, .001, ns | 23.24, .008, <.001 | .87, .000, ns | 4.03, .001, .045 | .597, .000, ns |
| Free lunch | 3.81, .001, ns | 1.56, .001, ns | 5.08, .002, .024 | 7.60, .003, <.01 | .031, .000, ns |
| Gender | 13.70, .005, <.001 | 2.01, .001, ns | 10.50, .004, <.001 | 3.93, .001, .048 | 11.08, .004, <.01 |
| Probable BN† | 14.43, .005, <.001 | 9.61, .003, <.01 | 15.81, .006, <.001 | 18.85, .007, <.001 | 3.61, .001, ns |
| Ethnicity | 1.98, .001, ns | 2.60, .002, ns | 2.07, .001, ns | 2.52, .002, ns | 3.33, .000, ns |
| Probable BN† x Gender | .003, .000, ns | 2.08, .001, ns | 2.37, .000, ns | .603, .000, ns | 3.81, .001, ns |
| Probable BN† x Ethnicity | 1.20, .001, ns | 1.49, .001, ns | .57, .000, ns | 1.80, .001, ns | .395, .000, ns |
| Gender x Ethnicity | 1.54, .001, ns | 2.08, .001, ns | 3.88, .003, <.05 | 4.59, .003, <.01 | .068, .000, ns |
| Probable BN† x Gender x Ethnicity | 1.30, .001, ns | 1.69, .001, ns | 3.82, .003, .022 | 33.77, .003, .023 | 2.57, .002, ns |

Probable BN† - Probable Bulimia Nervosa
