# Supplementary material for: Prevalence and early risk factors for bulimia nervosa symptoms in inner-city youth: gender and ethnicity perspectives
Source: J Eat Disord. 2021 Oct 21;9:136. doi: 10.1186/s40337-021-00479-5 (PMC8529812; doi:10.1186/s40337-021-00479-5)

Figures 1-10. Comparisons of the early risk factors scores by ethnicity and gender

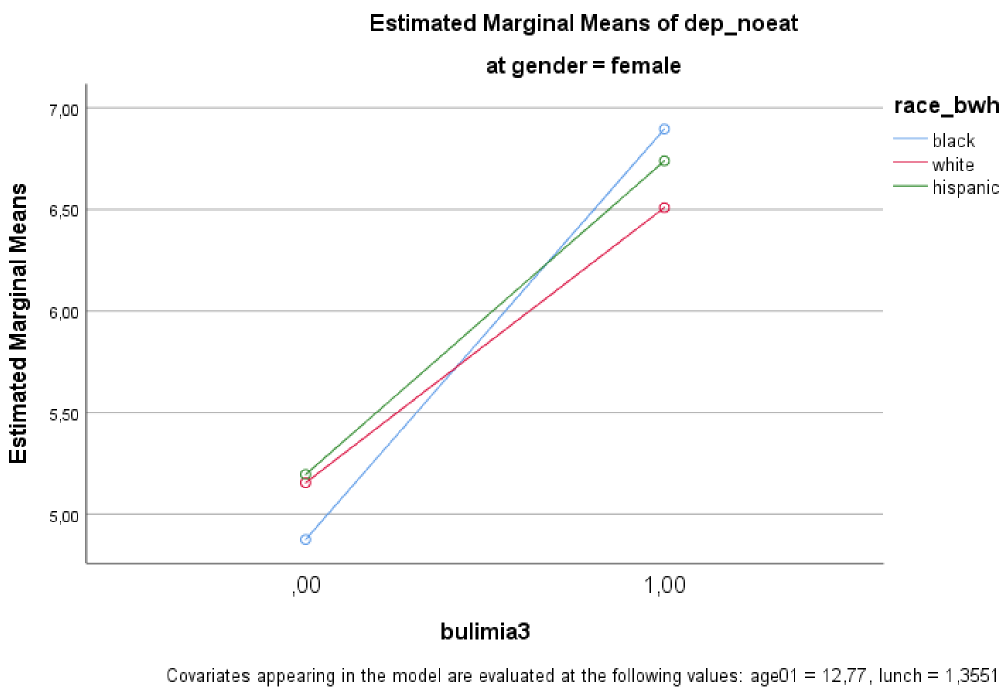

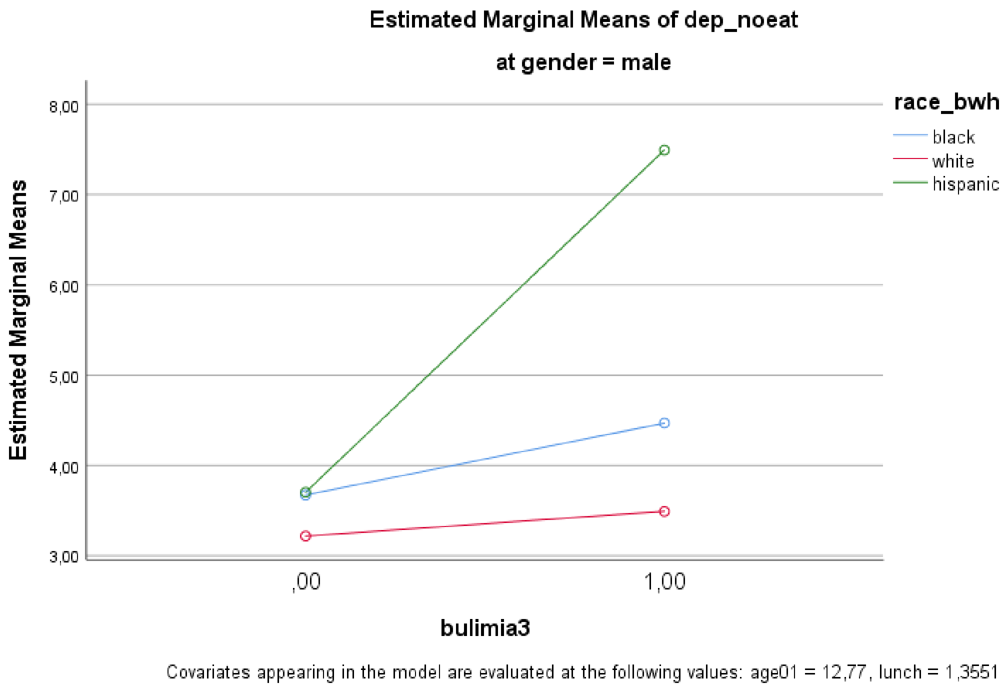

Figures 1-10. Comparisons of the early risk factors scores by ethnicity and gender

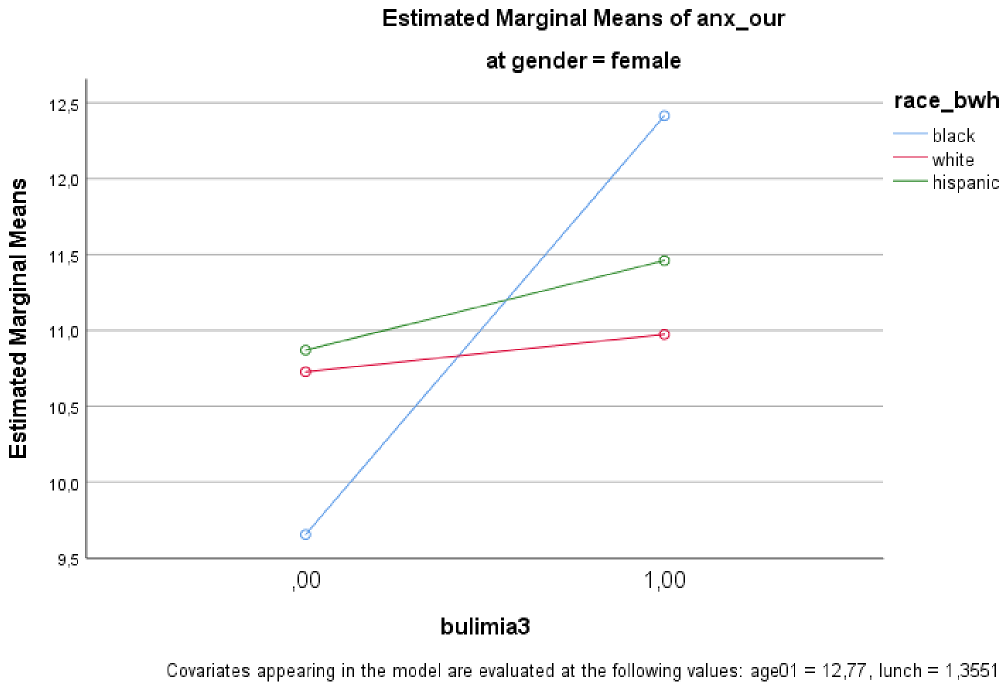

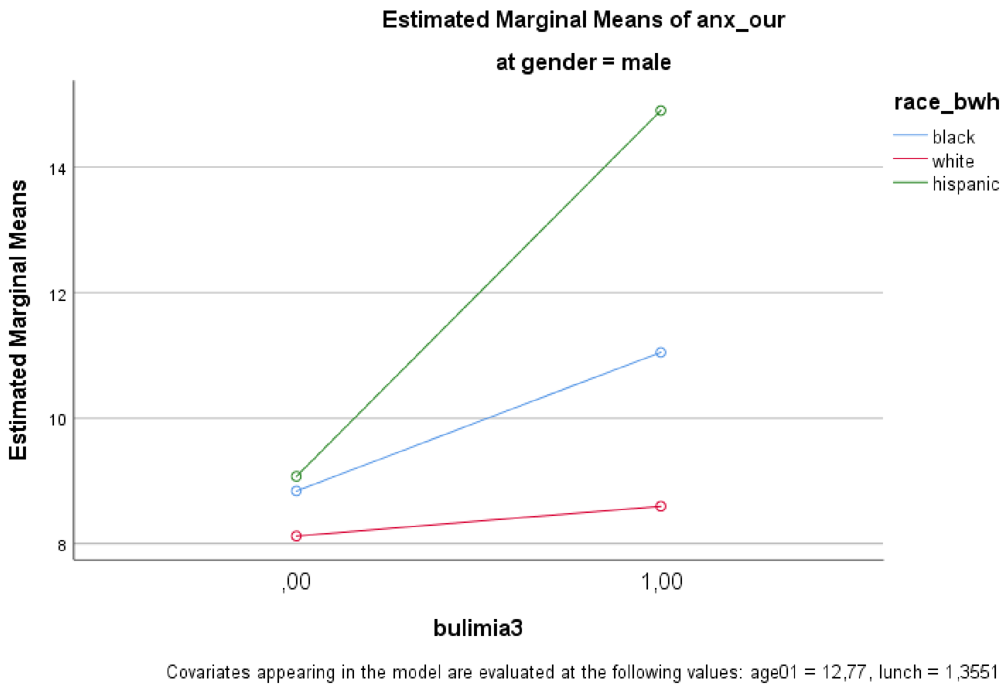

Figures 1-10. Comparisons of the early risk factors scores by ethnicity and gender

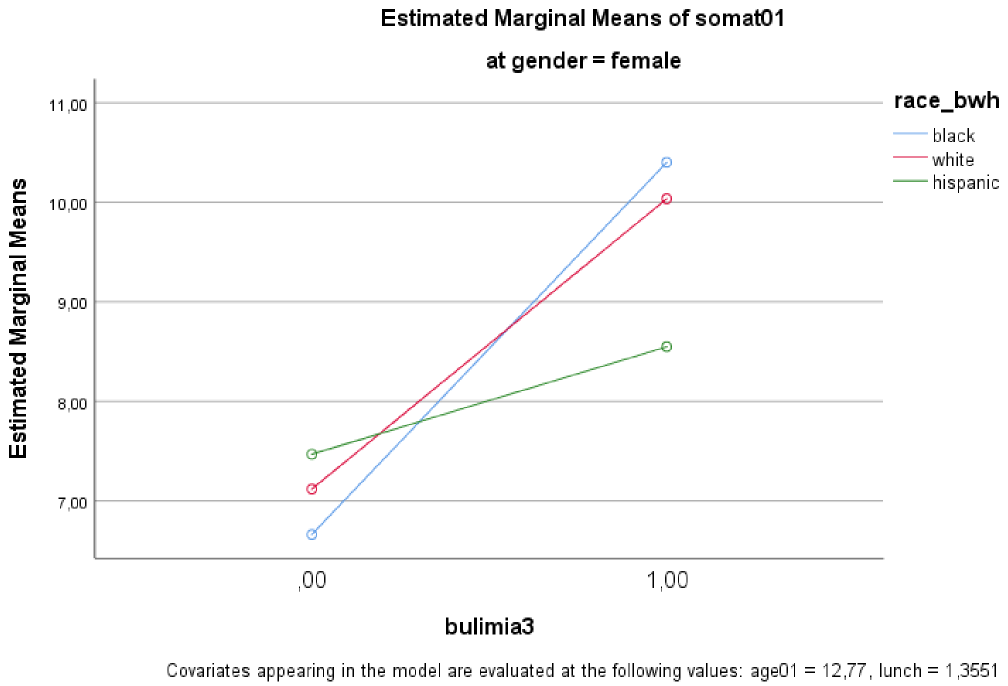

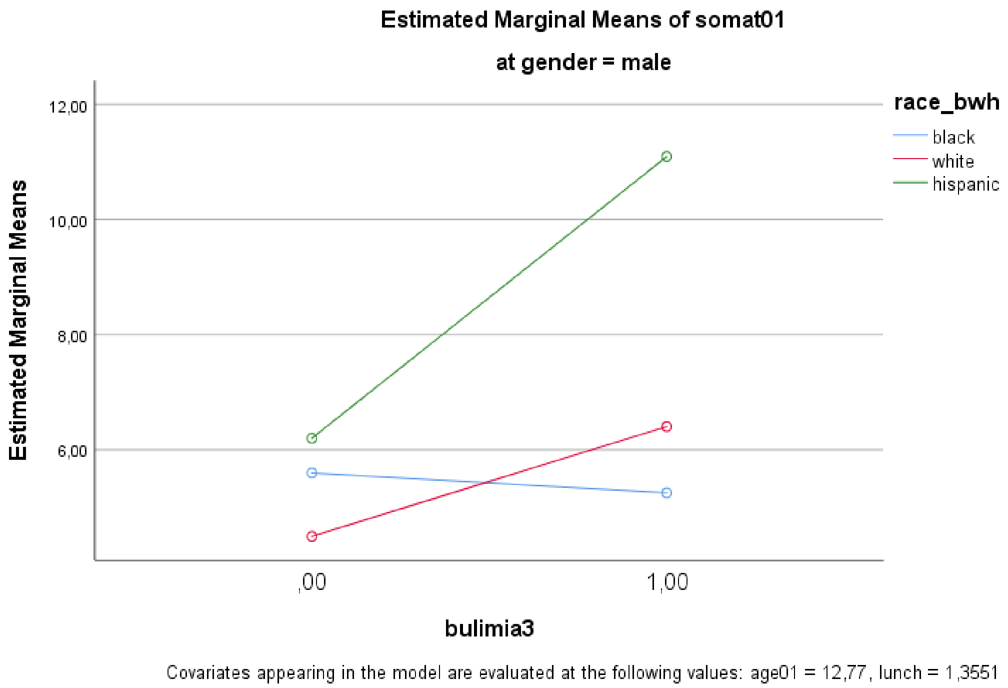

Figures 1-10. Comparisons of the early risk factors scores by ethnicity and gender

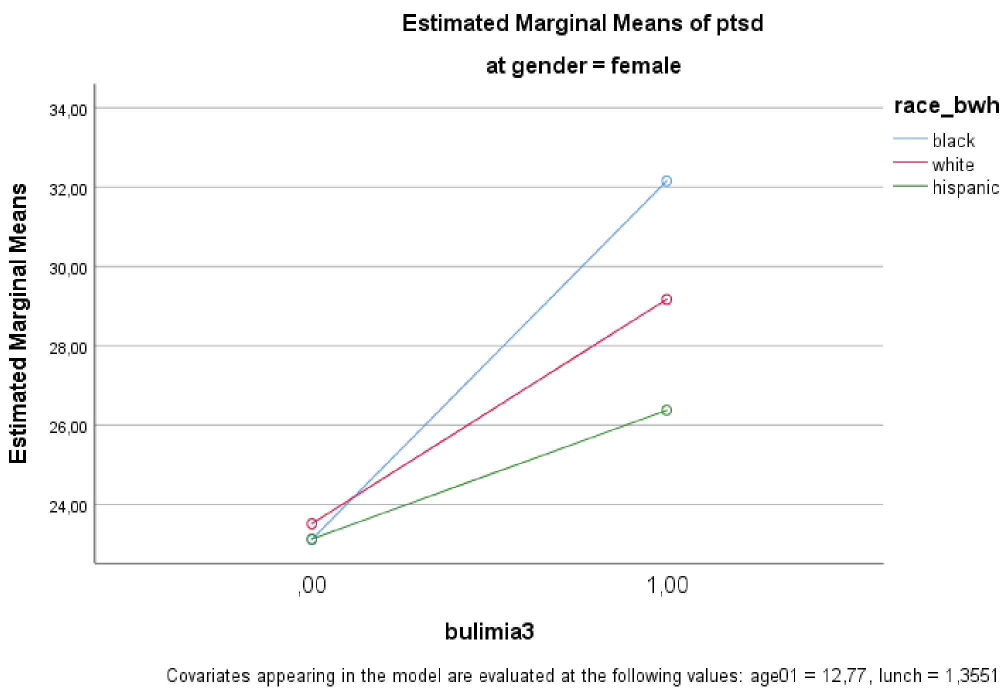

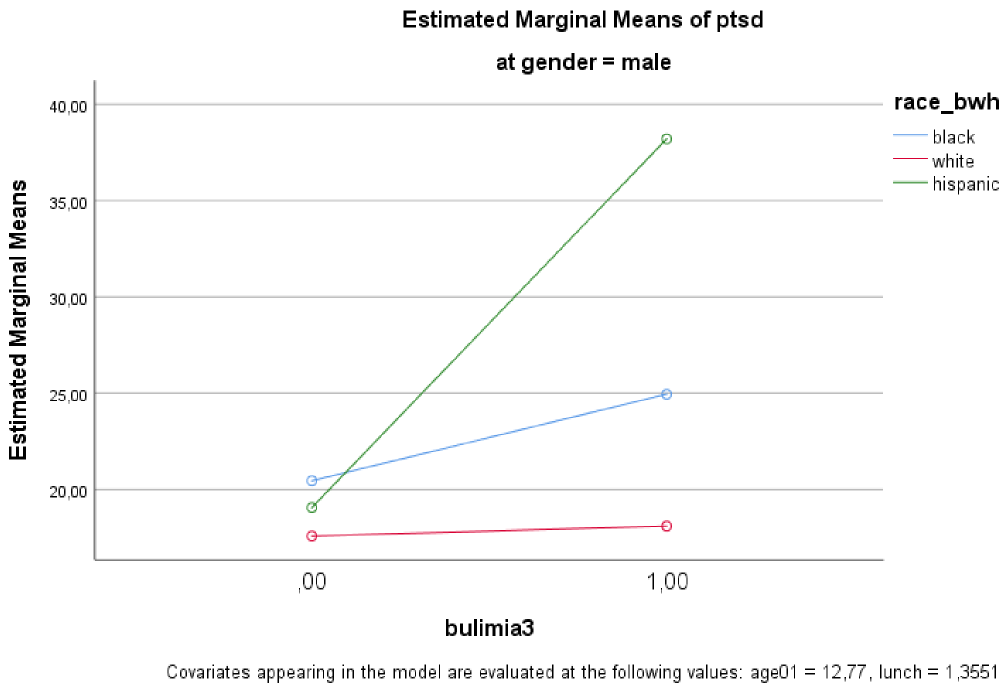

Figures 1-10. Comparisons of the early risk factors scores by ethnicity and gender

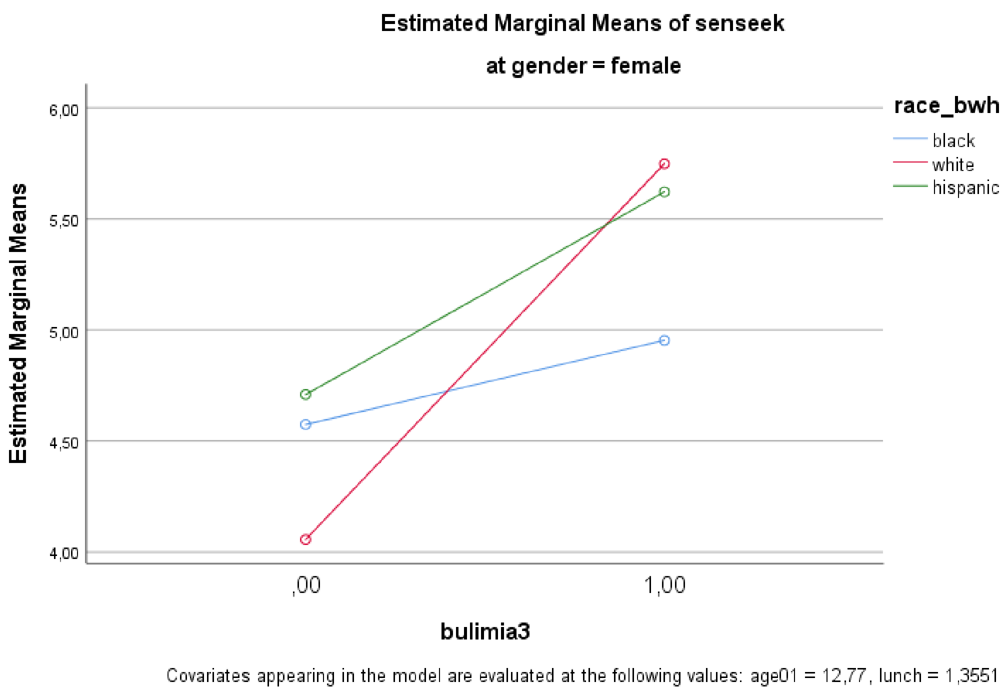

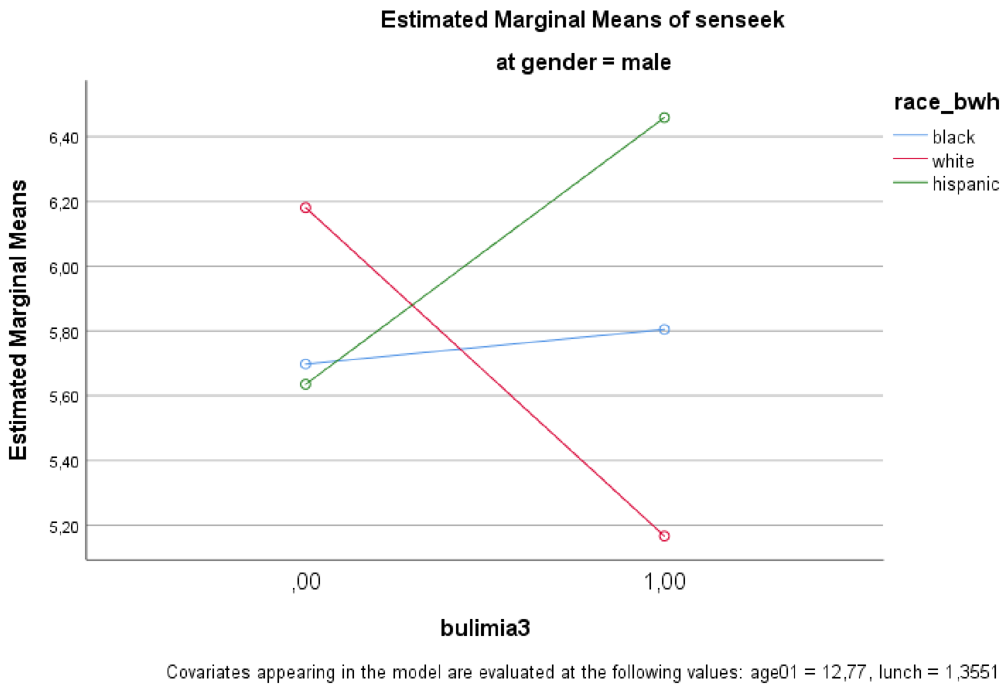

Supplement: Supplementary file 2 [file 40337_2021_479_MOESM2_ESM.rtf]
